# Persistent infection with high-risk human papilloma viruses: cohort study, Mérida, Venezuela

**DOI:** 10.3332/ecancer.2015.579

**Published:** 2015-10-08

**Authors:** Luis Téllez, Elvia Michelli, José Andrés Mendoza, Silvana Vielma, María-Eugenia Noguera, Diana Callejas, María Cavazza, María Correnti

**Affiliations:** 1 Los Andes University, Department of Microbiology, Mérida CP 5101, Venezuela; 2 University of Orient, Department of Bioanalysis, Sucre CP 6101, Venezuela; 3 Los Andes University, Department of Gynecology and Obstetrics, Mérida CP 5101, Venezuela; 4 University of Zulia (LUZ), Regional Reference Virology Laboratory, Maracaibo CP 4011, Zulia; 5 Institute of Biomedicine, MPPS, Caracas CP 10104, Venezuela; 6 Institute of Oncology and Haematology, MPPS, Caracas CP 1050, Venezuela

**Keywords:** human papilloma virus, polymerase chain reaction, persistent infection

## Abstract

Cervical lesions have been associated with infection by high-risk human papilloma virus (high-risk HPV). In 409 women aged >15 years high-risk HPV lesions were identified. In a cohort of this population persistent infection was compared with cytological, colposcopic, and histological lesions. Cervical scrapes were taken and DNA was isolated. HPV was detected by PCR in the E6/E7 region. Genotyping was performed by PCR nested multiple E6/E7. HPV was detected in a 37.40% (153/409), high-risk HPV in 86% (153/178), HPV18 46.64% (83/178), HPV16 34.28% (61/178). Among these 53.93% (96/178) were multiple infections, and HPV18/16 (30/96) was the most frequent 31.25%. The cytology showed changes in 15% of positive patients. A 49.67% in women positive for HPV infection showed abnormalities in the colposcopic study, a relationship that turned out to be statistically significant ( *p* < 0.0019 test χ^2^). Among all 85% of the women were younger than 45 years of age. Fifty-seven patients were evaluated 15 months after the base study, with initial prevalence of morbidity 49.12% (28/57) and at the end 10.53% (6/57), showing in 89.29% (25/28) negative for HR-HPV infection, 10.34% (3/28) showed persistence of infection, 17.54% (10/57) presented cytological alterations, with 80% of positivity for HPV, and a regression of 100% (10/10) of the previously identified lesions. With colposcopy, 50% (14/28) presented alterations related to HPV, of these 85.71% (12/14) showed regression of such an alteration. The cumulative incidence for HPV was 10.34% (3/29). The incidence rate was 4.23% (3/71), which is equal to 4.23 new cases of HPV infection per 100 people, per year of follow-up. In conclusion, the present work shows a high frequency of infection by high-risk HPV, with predominance of HPV18 and 16 and in general for multiple infections. Colposcopy was better predictor than the Pap smear for infection. The follow-up study revealed a low percentage of persistent infection, and a high frequency of negativity for viral infection, high regression of cytological and colposcopic lesions, a low cumulative and incidence rate similar to that reported by other Latin American countries and higher than the European countries.

## Background

Cervical cancer (CC) was associated with HPV infection in the 1970s, when concerns arose about the hypothesis proposed by Dr. Harold zur Hausen that this could be caused by infection with the virus identified in the condylomata acuminata [1]. In 2009, Dr. zur Hausen was awarded the Nobel Prize in Medicine for this novel idea, and for demonstrating the presence of the HPV genome in tissue from CC [2]. Cervical infection by high risk oncogenic HPV genotypes (high-risk HPV), is closely linked to the diagnosis of pre-invasive and invasive CC; it is reported that approximately 80% of these cases may be associated with infection by the genotypes of high risk HPV 16, 18, 45, and 31 [3].

Currently, CC represents the fourth most common type of cancer in the female population, constituting an important cause of morbidity and mortality, with an incidence of approximately 528,000 new cases and a cause of 266,000 deaths globally during the year 2012; with about 80% of these cases affecting women who reside in developing countries, where the CC totals 12% of all diagnosed neoplasms, and 7.5% of all deaths in the female population. Similarly, it has been determined that South America is included among the regions of greatest risk for the development of CC, with an age-standardised rate of 31.5/100,000 women [4].

Studies in the course of the last ten years have shown that the prevalence of HPV infection in our country is over 25%, both in women who are asymptomatic for cervical pathology and those that have a degree of diagnosed cytological abnormality [5–7]. According to statistics from the National Tumour Registry of the Ministry of Popular Power for Health (MPPS) of Venezuela, CC is the second cause of death in Venezuelan women between the ages of 15 and 76; it has also been reported that in our country every year approximately 4000 cases of CC are diagnosed and 1700 women die from this disease [8, 9].

The progression of lesions related to cervical infections by high-risk HPV as far as CC, occurs only in a small percentage of the affected women, because of the fact that these infections are self limiting, with the average time for resolution being 6–18 months, in those cases the woman does not show any clinical symptoms and the infected area is colposcopically and cytologically normal. A possible explanation of this approach is based on the fact that some HPV genotypes are more persistent than others, an ability that is genetically coded and therefore associated with this pathogenicity [2].

Persistent HPV infections have a high possibility of developing into cancer, if the associated genotypes are included in the oncogenic high risk group, due mainly to the fact that the persistence may induce secondary genetic changes, given the interference of the viral oncoproteins in the cell cycle checkpoints, and their ability to induce immortality in the keratinocytes [10–12]. In this sense, it has been reported that of patients with infections caused by high-risk HPV that persist for any length of time, approximately one-third of those with CIN·3 will progress to invasive CC in between 10 and 20 years, which is why these lesions should be the target of screening programmers for the prevention and early detection of CC.

In accordance with the concepts outlined above, it was considered important to conduct a clinical prospective epidemiological cohort study, aiming to establish prevalence of cervical infections because of HPV, demonstrate the possible relationship between high-risk HPV infection and the progression to different degrees of severity of the cytological and colposcopic lesions detected and to evaluate the persistence of the infection because of HPV and the cervical lesions by means of a cohort study, which would contribute to the epidemiology reality regarding the behaviour of the infection because of this viral agent, its impact on the health of women in our community, and the evaluation of the progression or resolution of cervical lesions associated with HPV.

## Methods

**Type of study:** This clinical epidemiological study followed the experimental design of cohort, being the prospective concurrent type.

Population sample: in order to carry out the investigation, a total of 409 women were selected at random all being between the ages of 15 and 69, sexually active or who had initiated sexual activity, and had attended the gynaecology department of the Autonomous Institute University Hospital of Los Andes (IAHULA) of Mérida state, Venezuela, during the time span in which the study was conducted.

**Inclusion and exclusion criteria:** Included in the investigation were women who had attended the gynaecology department and had undergone a semi-annual, annual, or bi-annual routine screening. Likewise, excluded from the study were all women who, for the time of the interview, and sampling, presented genital bleeding by normal menstruation, metrorrhagia or menorrhagia, and those who showed changes in the cervix because of surgical procedures that prevented proper sampling. Similarly women with diagnosed pregnancy and women who were sera-positive for HIV or undergoing immunosuppressive treatment were excluded.

**Bioethical standards:** To all the selected participants and to the parents and/or representatives of the minors, the nature, and importance of the study was explained and were told that it would be carried out following the principles laid down by the World Health Organisation for medical research in humans [14]; in addition, they were asked to give agreement by way of informed consent. The protocol of the study was evaluated and endorsed by the International Bioethics Committee of the Institute of Biomedicine, Ministry of Popular Power for Health and by the Council of Faculty of Medicine, ULA, Mérida.

**Follow-up protocol:** Among them 57 of the women participating in the study underwent a monitoring protocol during 15 +/- 5 months, distributed according to the reports of HPV detection, cytological and colposcopic, to assess the occurrence and/or evolution of possible pathological endocervical alterations, and their relationship to the incidence and persistence of HPV infections, identified in this group of patients.

**Sampling:** All participants underwent a gynaecological evaluation, which was carried out by qualified medical personnel; with the report of this study being recorded in a clinic registered designed for that purpose.

Cervical swab samples were obtained for later processing using the Papanicolaou technique. The results are expressed according to the terms of the Bethesda classification, namely: negative for intraepithelial lesions or malignancy; atypical squamous cells (ASC), including cells with alterations that are not clearly pre-cancerous; atypical squamous cells of undetermined significance (ASC-US), including all the cells that are among the cellular changes benign and intraepithelial lesion; atypical squamous cells that do not exclude a high grade lesion (ASC-H); low grade intraepithelial lesion (LEL BG), including cases of mild dysplasia/NIC I, and the cellular changes associated with HPV infections; High-grade squamous intraepithelial lesions (HSIL), cellular changes associated with moderate or severe dysplasia/CIN 2, CIN 3, and carcinoma *in situ* (CIS) [15].

A second cervical specimen was taken with a swab, using the DNA collection device kit (Digene® Corporation, Gaithersburg, MD, USA); which were used for the isolation of DNA and the identification of HPV cervical infections.

**Sample of cervical biopsies:** The cervix biopsies of the 41 patients who had cervical lesions at the time of the gynaecological examination, were obtained by the gynaecologist; a portion of the tissue was placed in 10% buffered formalin for histology analysis, and another portion was placed in a ATL lysis solution (QIAGEN®), and were frozen at −70 °C until required for processing. The results are expressed according to the terms of the classification established by the National Cancer Institute in the USA (NCI, the English initials of the National Cancer Institute). This assessment was carried out using staining with haematoxylin–eosin (H&E) to the tissue samples, which helps to demonstrate the presence of histopathologic lesions. The NIC is divided into grades I, II, and III. NIC I corresponds to the mild dysplasia, CIN II to the moderate dysplasia, and CIN III to the severe dysplasia and cancer *in situ* [15].

**Colposcopic evaluation:** All the participants in the study underwent a colposcopic exam of the cervix, vagina, and vulva. The international nomenclature established by the International Federation of Cervical Pathology and Colposcopy (IFCPC) was used, to classify the patterns observed colposcopic studies [16]. According to this classification, the patients were placed in the following categories: normal colposcopic findings; abnormal colposcopic findings, which included all the atypical observations, which are further divided into major and minor colposcopic abnormalities. The minor atypical flat acetowhite epithelium, fine mosaic, fine punctuation, and epithelium iodine partial positivity. On the other hand, the major abnormalities include dense acetowhite epithelium, coarse mosaic, coarse punctuation, iodine negativity, and atypical vessels; colposcopic features suggestive of invasive cancer; unsatisfactory colposcopy, and miscellaneous findings (condylomata, keratosis, erosion, inflammation, atrophy, deciduosis, and polyps).

The colposcopic evaluation of the patients was included as a tool for the routine diagnosis of cervical atypia, in accordance with the parameters proposed for Venezuela in the Consensus Meeting on Human Papilloma Virus, 2008, published in the Caracas Medical Gazette [17].

### Processing of samples for the diagnosis of HPV

**Isolation of DNA:** The commercial kit for the QIAamp DNA Mini Kit (QIAGEN®) was used in accordance with the instructions of the manufacturing laboratory.

**Quantification of the extracted DNA:** The concentration of the extracted DNA was determined by spectrophotometry at 260 nm, using the UV photometer T1101/1101 (Biotech, Cambridge, UK).

**Figure 1.**
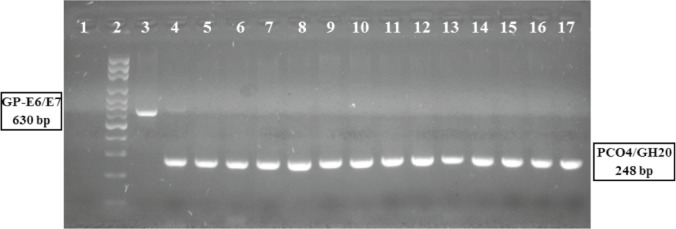
Polymerase chain reaction assay, amplification of HPV viral gene region E6/E7. HPV PCR E6/E7 assay: DNA from cervical samples was amplified by PCR (see methods). 1: negative control; 2: molecular weight ladder; 3: Generic positive control HPV-C001, amplicon of 630 pb; 4: HPV positive sample; 5–17: HPV negative samples. All samples shower the amplification of specific fragment to human beta-globin gene (4–17).

**Amplification of DNA fragments of HPV specifics by multiple nested-PCR, viral gene region E6/E7:** This technique was applied to the detection and genotyping of HPV. The first reaction allowed the detection of specific sequences of HPV DNA, contained in a conserved region of the early genes *e6/e7*, using the pairs of consensus primers GP-E6-3F/ 5B/ 6B, according to the protocol previously described by Sotlar *et al*, 2004 [18]. As an internal control of the reaction a fragment of approximately 248 bp of the b-globin human gene [19] and for the DNA HPV positive control the commercial oligonucleotide, HPV-C001 (Maxim Biotech, Inc) was used. All the reactions were carried out in a final volume of 25 μL, containing 100 ng of total genomic DNA, 10 μL of HotStarTaq® Master Mix 2X, and 400 nm of each oligonucleotide (Figure 1).

**Figure 2.**
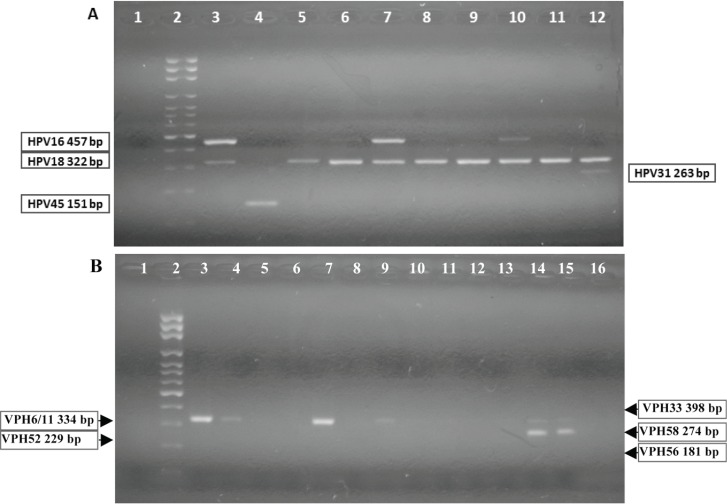
Nested-polymerase chain reaction-multiplex assay, amplification of HPV viral gene region E6/E7. Nested-PCR-multiplex assay: DNA from cervical samples was amplified by PCR (see methods). A: identification to HPV16, 18, 31, 45. 1: negative control; 2: molecular weight ladder; 3: generic positive control HPV-C001, amplicon of 457bp (HPV16 positive control); HPV-4009-11-18, amplicon of 322bp (HPV18 positive control); 4: HPV 45 positive sample; 5, 8, 9, 11: HPV 18 positive samples; 6, 7, 10: HPV16/18 positive samples; 12: HPV18/31 positive sample. B: identification to HPV 6/11, 33, 52, 56, 58. 1: negative control; 2: molecular weight ladder; 3: HPV-4009-11, amplicon of 334bp (HPV11 positive control); 4, 7, 9: HPV6/11 positive samples; 14, 15: HPV6/11/58 positive samples; 6, 8, 10–13, 16: HPV negative samples.

The multiple PCR, nested format, allowed for the identification of the high-risk oncogenic viral genotypes HVP16, 18, 31, 33, 45, 52, 56, and 58, and low-risk HPV6/11 [18]. In this test for the reaction mixture 10 μL of HotStarTaq® Master Mix 2 X and 400 nM of each oligonucleotide was used; as target DNA, 2 μL was taken from the 1/10 dilution of the product of the PCR GP-E6/E7, in a final volume of 25 μL. For HPV positive controls: in each reaction commercial oligonucleotides in DNA-HPV, HPV-C001 for VPH16/18, HPV-4011-18 for VPH18 and HPV-4012-11, to VPH11 were used all from Maxim Biotech, Inc. The other identified genotypes were evaluated from the molecular weight of the amplicon obtained in the PCR, according to their location in the agarose gel, in comparison with the molecular weight marker used (Figure 2).

All of the previously described amplifications were processed in a thermal cycler model ABI 2400 (Applied Biosystems). To view the reaction products, these were subjected to electrophoresis in agarose gel to 1.20%, with 10 μL of ethidium bromide/100 mL of agar, and rear illumination with UV light. The electrophoretic run included a marker of molecular weight of 1000 pb, ladder of 100 BP (100 bp DNA Ladder, Invitrogen), and the buffer 10 X blue juice TM loading gel buffer (Invitrogen) to verify the size of the bands obtained.

**Definition of persistent HPV infections:** For a persistent infection positivity for the same viral genotype was taken as the same persistent infection on two specific occasions: in the first assessment and one that took place 15 months later. It is important to note that all the patients included in the follow-up were said to be sexually active; which is why the ‘sexual abstinence’ was not assessed.

**Collection and analysis of the data:** A database in the EPI Info 2012 programme, version 7.0 was created, which was used for the descriptive statistical analysis of the results, frequency distribution of infections by HPV of high and low risk and association in qualitative scales of the different groups identified, with clinical and epidemiological variables from patients, which were summarised in proportions. Inferential statistical analysis and the definition of significant associations were conducted using the Chi-square test. The indicators rate of incidence, or incidence density, calculated as the quotient of the number of new cases of disease which occurred during the monitoring period and the sum of all the individual observation times and accumulated incidence, referred to the proportion of candidates who developed the disease during the observation period.

## Results

### Frequency of human papilloma virus infection detection

Of 409 women studied, HPV DNA was detected in 37.40% from cervical specimens (Table 1).

### Frequency distribution of infection by HPV by age groups

**Figure 3.**
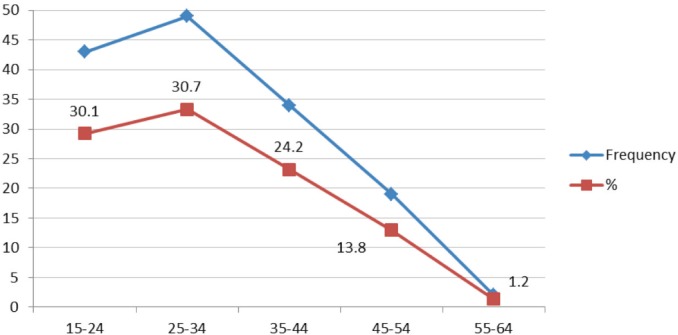
Frequency of HPV detection in cervical samples from feminine patients for age groups, Mérida- Venezuela, 2008–2012.

Source: Microbiology and Public Health Laboratory. Faculty of Medicine. ULA. MPPS. Mérida.

85% of HPV infections arose in women under 45 years of age; there was a similar percentage in women 15 to 24 years of age and 25 to 34 years (Figure 3).

### HPV genotype prevalence found in the 153 samples positive for infection

Among these 85.96% of HPV found corresponded to high-risk oncogenic genotypes, from those 80.92% were genotypes HPV16 and 18, with a percentage of 34.28 for the first and 46.64 for the second (Table 2).

### Frequency of single and mixed infections by HPV genotype found in 153 cervical samples

From all of the genotypes found, 53.93% (96/178) correspond to mixed infections. The most frequent combinations were HPV18/16 with 31.25% (30/96) and HPV18/6y11 with 20.83% (20/96), the later with statistical significance ( *p* < 0.000029 test χ^2^). However; when considering each genotype separately, both HPV18 and HPV16 showed greater frequency for individual infections, 58.54% (48/82) and 37.80% (31.82) respectively (Table 3).

## Relationship between infection from HPV and cytological results in cervical samples

**Figure 4.**
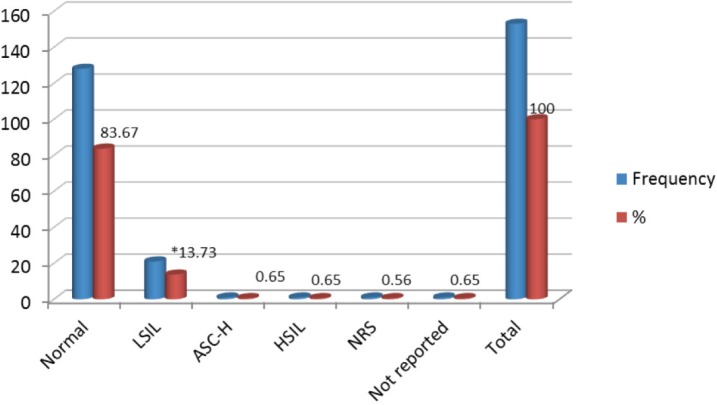
Relationship between HPV infection and cytological diagnosis in cervical samples, Mérida-Venezuela, 2008–2012.

^*^LSIL and HPV18 infection (*p* = 0.02; χ^2^); NRS: not representative sample.

Source: Microbiology and Public Health Laboratory. Faculty of Medicine. ULA. MPPS. Mérida.

From 153 samples positive for HPV infection, 83.67% (128/153) showed normal cytological results, while 13.74% (21/153) were reported as LSIL; other categories such as HSIL and ASC-H were reported in very low percentages of 0.65% for each one. When comparing the LSIL cytological report with infection from HPV18 there was an association with statistical significance found ( *p* < 0.02 test χ^2^) (Figure 4).

### Relationship between HPV infection and colposcopy results of cervical samples

**Figure 5.**
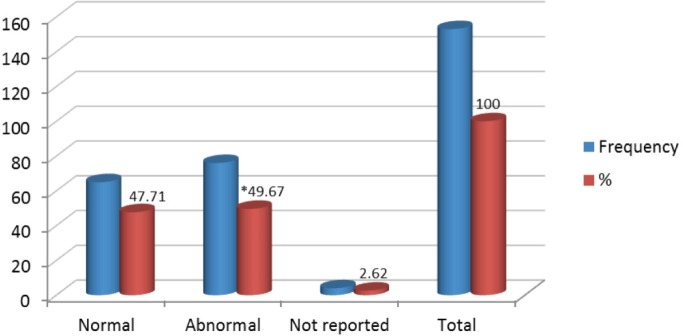
Relationship between HPV infection and colposcopic diagnosis, Mérida-Venezuela, 2008–2012.

^*^HPV and abnormal colposcopic findings (*p* = 0.0019 χ^2^)

Source: Microbiology and Public Health Laboratory. Faculty of Medicine. ULA. MPPS. Mérida.

**Figure 6.**
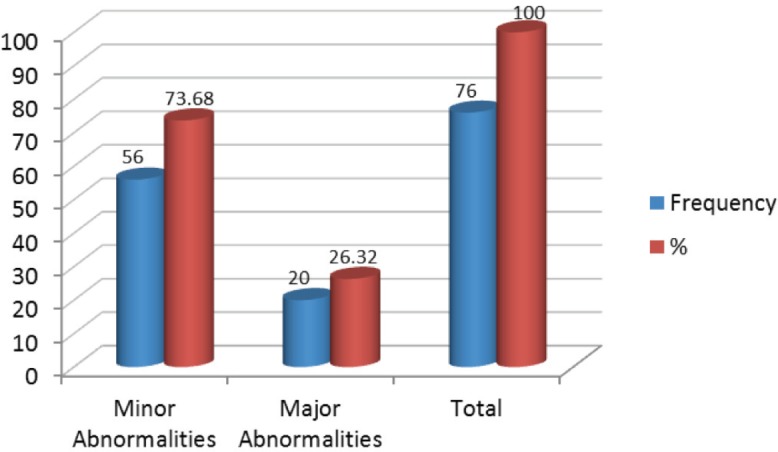
Relationship between HPV infection and colposcopic abnormalities diagnosed, Mérida-Venezuela, 2008–2012.

Of 153 samples positive for infection with HPV, 49.67% (76/153) showed abnormal colposcopy results and 47.71% (73/153) had normal colposcopy results. Within the abnormal results 96.05% corresponded to minor colposcopy atypia and 26.32% to major colposcopy atypia. Significant statistical association between infection from HPV and abnormal colposcopy results was observed, especially within the TZ (transformation zone) and the iodine-negative clear boundary area ( *p* < 0.0019 test χ2). And also for dense and thin acetowhite epithelium ( *p* < 0.0019 test χ^2^). The association between infection from HPV and a positive Schiller’s test resulted statistically positive ( *p* < 0.02 test χ^2^) (Figures 5 and 6).

## Persistence in the diagnosis of infection from HPV and its genotypes in cervical samples from 57 patients who participated in a monitoring study

When following-up with a cohort of 57 women of the initial sample, for a period of 15 months, an initial prevalence of 49.12% (28/57 women) morbidity was observed, based on this condition, in the second phase of the study, there were findings of high negativisation of the infection, displayed by the absence of HPV DNA detection in the cervical samples of 89.29% (25/28) of the women initially infected with HPV; likewise, there were findings of low prevalence of HPV infection, 10.71% (3/28 women) and after studying the prevalence of specific genotypes, there was just one case found for HPV18 representing 16.67% (1/6) of the patients who were initially positive for this viral genotype. These observations allow the establishment of an accumulated incidence of HPV infection of 10.34% (3/29) in 15 months. Upon determining the level or density of viral infection, a value of 4.23% (3/71) was obtained, which is equivalent to 4.23 new cases of HPV infection per 100 people, per year of follow-up (Table 4).

### Prevalence in the detection of HPV infection in cervical samples of 57 patients who participated in a follow-up study and its relationship with the cytological report

In the initial sample, 17.54% (10/57) of the women evaluated, presented cytological alterations, and 80% (8/10) of them were positive for the detection of HPV DNA. Upon observing the results obtained after follow-up, there was a regression of 100% (10/10) of the previously diagnosed lesions in all patients, both positive and negative for HPV, and negativisation of HPV infection. Two patients presented new cervical lesions (LSIL in both) after time of follow-up; it is important to point out that these two patients presented new HPV infections, classified in the group of those which resulted negative for the viral genotypes researched in this study (Table 5).

### Prevalence in the detection of HPV infection in cervical samples of 57 patients who participated in a follow-up study and its relationship with the colposcopy report

Among the 50% (14/28) of the positive patients’ for HPV infection in the initial sample showed colposcopy lesions with few differences between the cases with minor and severe atypia. In 85.71% (12/14) of those cases, there were observations after the monitoring timeframe, of the disappearance of the initial colposcopy alterations that were found, and there was negativisation of the HPV infection (Table 6).

## Discussion

The percent of viral infection detection was 37.40%, pointing out that 83.67% of these HPV positive reports were from women with normal cytology, this is a finding which is considered superior to that registered in the general population around the world, making up between 9–13% [3, 9, 21]; in addition, the percentage of determined HPV infections contrasts with the estimate described previously by Bruni *et al* [22] for Latin America, which describes a prevalence of a 16.10% of HPV in women with normal cytology.

The high percentage of HPV positive cervical samples found in our study could be because of the nature of these infections, which have displayed high variability, depending on multiple factors, such as demography, behavioural characteristics of the population being studied, and the method of diagnosis being applied [23]. In the same manner, studies on the subject carried out in Venezuela express a variable positivity range for this infection [5, 24–27]. It is in the sense that when contrasting the findings of the present study with two other studies done with the female population of the state of Mérida, using diagnosis protocols different from those used in this study (HPV PCR-E6/ E7) so to say a second-generation hybrid capture test was applied in one of them [28] while the technique for amplification of specific genetic sequences PCR-RFLP [29] was used in the other [29], there was a prevalence of HPV of 12.54% and 51.90% respectively. In addition both in our study and in the first of the mentioned studies, women with and without previous HPV infection were evaluated. In the second instance only women with a previous clinical diagnosis of viral infection were studied, whether it was through cytology, colposcopy, or biopsy. This data suggests that the diagnostic method used and the characteristics of the studied population have a direct influence on the level of viral infection detection.

It is important to emphasise the high levels of HR HPV genotypes identified in the population studied in this investigation, both in multiple and single infections; in addition, it is important to point out that HPV18 was the most frequently detected viral genotype, followed by HPV16. The relative distribution of these genotypes in the studied women is similar to the data previously published in the city of Mérida, Venezuela, which shows a predominance of HPV18 infections over all genotypes studied [28]. On the other hand, the results in the present study differ from the information previously published by other researchers around the world, where HPV16 was the most frequently identified genotype [30–33]; suggesting that the reported HPV genotypes in regions around the world could vary in type and relative incidence [33].

Within the samples positive for HPV DNA identified in this study, multiple infections with two or more genotypes were reported in 53.93% showing the importance of genotyping evaluations in patients with a positive report for viral detection. This relevance is based mainly on the fact that multiple infections caused by different HPV genotypes increase the probability of acquiring a high-risk genotype [23]. In addition, these co-infections have a high level of occurrence, which suggests the presence of a synergistic action among the different viral genotypes diagnosed in a single sample [34]. In other research done on the subject, there is support that clinical epidemiological implications of infections with multiple genotypes of HPV are uncertain. This claim is based on the follow-up study done on women with multiple HPV infections, in which there were observations that even though the identified genotypes tend to form groups, the monitoring of the infection in these women does not show that these groups have a determining influence in the course of the viral infection and the development of malignant lesions in the affected patients [35].

The results obtained in the present study, based on cytological analysis of the cervical samples through the Pap smear technique, show that 15.03% of HPV positive patients showed cytological changes suggestive of this viral infection, emphasising the predominance of infections by HR HPV in patients with a diagnosis of LSIL. The previously presented data reflects a relationship between identified HPV genotypes and the abnormal cytological report. Consequently, these results support the widely known concept regarding the importance in the detection and genotyping of HPV in the routine gynaecological consultation. With this in mind, previous publications have shown an elevated risk of progression of cervical lesions, from CIN 1 to CC, in patients infected with HPV16 and/or HPV18; with an estimated timeframe of 12 months, in approximately 3.5–12% of the women infected with a HR HPV genotype, there occurred progression to cervical lesions of different grades of CIN (12, 34). Similarly, two meta-analysis in publications with world prevalence of HPV infection and the distribution of viral genotypes in cervical samples of women with normal and abnormal cytological results concluded that the testing for diagnosis of HPV, simultaneous with the cytological evaluation, was associated with a significant reduction in the progression of cervical lesions to CC and in deaths because of this pathology [22, 36].

On the other hand, it is currently well-known that in about 60% of women with normal cytology and a positive HPV DNA test, there is negativisation of the viral infection report in the following six months from the first report. However, when dealing with patients over the age of 40, the risk of progression from CIN 1 to CIN 3 in the following ten years increases significantly, compared with that of young women [12, 37]. The variation of distribution of HPV infection in patients with SIL and CC, as well as the supported information in prospective studies, establishes that there is a genotype-specific risk in the development and evolution of cervical lesions in women with abnormal cytology; suggesting that in clinical practice the distinction of HPV genotype involved in the infectious process has the potential to improve the monitoring of women with SIL [22]. In other words, the identification of HPV has shown greater sensitivity in suggesting the presence of neoplasm lesions, compared to the Pap smear test [38].

It is important to mention the recommendations for CC screening tests issued by the American Congress of Obstetricians and Gynaecologists in 2012 which indicate that gynaecological evaluations with cervical cytology should be no later than one year; however, the time between evaluations could increase to an interval of 3–5 years with the realisation of cytology and the incorporation of screening tests for HR HPV infection [39]. This suggestion for screening is directed at decreasing the rate of CC that is recorded yearly around the world, especially in developing countries, since conventional cytology has limitations when comparing its sensitivity and specificity with that of colposcopy, or histopathological, or biopsy analysis [37].

The colposcopy exam done suggested the viral infection in a range superior to that obtained through the Pap smear technique, and in addition there were observations of a significant statistical association between the detection of HPV and the abnormal colposcopy findings, showing that the colposcopy had high predictive value in the diagnosis of HPV infection. These results coincide with those reported by Mitchell *et al* [40], who found an average sensitivity and specificity of 96% and 48% respectively, in the colposcopy for the detection of abnormal histopathologies, including CIN 1 to CIN 3 and CC. The findings of the present study confirm the importance colposcopy evaluation done to patients during a routine gynaecological exam; in that sense it has been shown that approximately 69% of women who present abnormal colposcopic changes are identified with at least one HR HPV genotype [41].

In the present study, 57 participants were monitored for a period of 15 months, with morbidity rates of HPV infection of 49.12% in this group. At the end of the monitoring period it was observed that the prevalence of positive cases for HPV infection was low (10.71%); likewise, the infection persisted in specific viral genotypes; it was only identified in one patient positive for HPV18 (16.67%). The data obtained in the present study coincides with that reported in another study, in which it was shown that infection by oncogenic HPV genotypes has an average duration of eight months, while the duration of non-oncogenic HPV has an estimated duration of four months [42]. Numerous studies have pointed out that persistent HPV infection is of extreme importance in the development and progression of precancerous lesions to CC, and that this process could take 1–10 years [13]. Likewise, it is known that the association between infection from HR HPV and the presence of CIN is stronger for a persistence of 12 months than for a persistence of six months, although this relationship could vary depending on the viral genotype [43]. Some data suggests that HPV16 persist on an average more time infecting the cervical epithelium, in comparison with other viral genotypes, thus lesions associated with the presence of HPV16 could progress to cancer quicker than those that do not present HPV or have another viral genotype [44].

Data complementary to that obtained in the present study regarding the prevalence of HPV infection shows that a generally high percentage of negativisation of the viral infection (89.29%) was determined in the 28 patients positive for HPV in the initial sample. In that sense, there is consistency between our results and the estimates of various studies done on women who were HPV positive, in which the resolution rate of the viral disease was high throughout the first year of infection as described in the work of Schmeink *et al* [45] and Muñoz *et al* [46]. They reported a resolution of the viral infection of 70% before the sixth month of the monitoring phase and an average duration of HR HPV infection of 14.8 months respectively.

The cumulative incidence of HPV obtained in this study (10.34% in 15 months) differs from studies previously published in which this indicator is considerably higher, Winer *et al* [34] with a reported cumulative incidence of 32.3% (confidence interval (CI) 95% = 28, 0-37, 1) after two years of monitoring, in 603 university female student; this difference could be because of the fact that the mentioned study included a greater number of participants and only young women, while in the present study the number of women were less and both young and older women were incorporated with an age range of 15 to 69. Muñoz *et al* [47] reported an elevated incidence of HPV infection in their follow-up study done on 1728 women during nine years. In this case, even though the studied population included women of a mature age (ranging from 15–85 years) the differences with our study would be defined by the number of participants and the timeframe of monitoring.

With regard to the rate or density incidence of the viral infection, there was an obtained value of 4.23 new cases of HPV per 100 people, per year of monitoring; these results coincide with those reported in another cohort study done in Colombia, which included 1610 women between the ages of 15–85, with abnormal cytology, who were monitored every six months for an average of 4.1 years, and the rate of incidence determined for HR HPV was five cases per 100 women a year [46]. In addition, Font *et al* [48], in 1383 women cared for in the metropolitan area of Barcelona, Spain, there were observations of an incidence of new infections of 2% per year throughout the monitoring of three years. The distribution of identified HPV infections in the 57 patients who underwent the monitoring protocol according to the results of the cytological evaluations that were done indicated that in the initial sample 17.54% of the participants presented cytological alterations, and after follow-up there was a regression of 100% of these lesions, both in women who were positive and negative for HPV. This regression was simultaneous to the viral negativisation, which supports the causal relationship between HR HPV infection and the occurrence of cervical lesion in the affected women.

The evaluation of persistent HPV infections, diagnosed in this study, according to the reported colposcopy atypia, showed that the three patients with this condition did not present colposcopy atypia after follow-up. On the other hand, there was regression of the colposcopic alterations at an elevated percentage in the patients who presented negativisation of the viral infection. The previously presented results support the concept under which the severity of the colposcopic alterations could be related to the frequency of persistent cervical infections caused by HR HPV [49, 50], a fact supported in that the majority of CCs are preceded by long periods of HR HPV infections, and the presence of these viral genotypes in the affected cells leads to multiple changes in the cervical intraepithelial tissue which increases the possibility of its progressive development to CIN 3 and to CC [49]. With regard to age, when considering infections positive for HPV, 85% of them were in women younger than 45 years of age, with maximum percentages of positive results in the group younger than 35 years of age. Different studies carried out in Venezuela report results that coincide with those obtained in the present study [5, 28, 51]. Infections positive for HPV decreased as age of the participants increased. This data coincides with findings obtained in work recently carried out in Italy, in which the prevalence of HR HPV genotypes had a significant increase in women younger than 35 years of age to later decrease as age of the participants increased [52]. The decrease in prevalence of HPV infection as age increases is frequently defined by changes in sexual behaviour and elimination of the infectious process mediated by the localised action of the elements of the immunological system of the affected person [53].

## Conclusions

An increased frequency of high-risk HPV infection was shown in the population of women under 45 evaluated in this study, with a predominance of HPV18 and 16 and in general multiple infections, a colposcopy was found to be a better predictor for HPV infection than cytology, with statistical significance between infection by this virus and abnormal colposcopy. The follow-up study of the cohort showed a low percentage of persistent infection, a high frequency of negativisation of viral infection, a high percentage in the regression of cytological and colposcopic lesions, a low cumulative frequency, and a similar incidence rate to that reported by other Latin American countries and higher than that of the European countries.

## Conflicts of interest

We declare that no conflicts of interest exist among the authors of this original research article.

## Figures and Tables

HPV and flat/dense aceto-white epithelium ( *p* < 0.0019; χ^2^).

Source: Microbiology and Public Health Laboratory. Faculty of Medicine. ULA. MPPS. Mérida.

**Table 1.** Frequency of HPV molecular detection in women, Mérida- Venezuela, 2008–2012.

HPV	Number	%	Accumulated %
POSITIVE	153	37.40	37.40
NEGATIVE	256	62.60	100.00
TOTAL	409	100.00	100.00

Source: Microbiology and Public Health Laboratory. Faculty of Medicine. ULA. MPPS. Mérida.

**Table 2.** Frequency of HPV genotypes identification in 153 cervical samples, Mérida- Venezuela, 2008–2012.

HPV	Frequency	%	Accumulated %
18	83	46.64	46.64
16	61	34.28	80.92
31	2	1.12	82.04
45	2	1.12	83.16
52	2	1.12	84.28
56	1	0.56	84.84
58	2	1.12	85.96
6/11	25	14.04	100.00
TOTAL	178	100.00	100.00

Source: Microbiology and Public Health Laboratory. Faculty of Medicine. ULA. MPPS. Mérida.

**Table 3.** Frequency of HPV genotypes as individual and multiples infections in 153 cervical samples, Mérida- Venezuela, 2008–2012.

HPV	Individuals infections	%	Multiples infections	%	Total
18	48	58.54	35	36.47	83
16	31	37.80	30	31.25	61
31	0	0.00	2	2.08	2
45	0	0.00	2	2.08	2
52	1	1.22	1	1.04	2
56	1	1.22	0	0.00	1
58	1	1.22	1	1.04	2
6/11	0	0.00	25^*^	26.04	25
TOTAL	82	100.00	96	100.00	178

Source: Microbiology and Public Health Laboratory, Faculty of Medicine, ULA, MPPS, Mérida.

**Table 4.** HPV initial infection, persistence, and negativisation of cervical infection in the cohort of 55 patients studied, Mérida-Venezuela, 2008–2012.

HPV genotyping	HPV initial infection		HPV infections persistence at 15 +/- 5 months		HPV infections negativisation at 15 +/- 5 months	
Individuals infections	*n*	%	*n*	%	*n*	%
HPV16	9	32.14	–	–	9	100.00
HPV18	6	21.43	1	16.67	5	83.33
Multiples infections						
HPV16/18	4	14.29	–	–	4	100.00
HPV18/6/11	2	7.14	–	–	2	100.00
HPV18/58	1	3.57	–	–	1	100.00
Others HPV genotypes	6	21.43	2	33.33	4	66.67
TOTAL	28	100.00	3	10.71	25	89.29

Source: Microbiology and Public Health Laboratory. Faculty of Medicine, ULA, MPPS, Mérida.

**Table 5.** Correlation between HPV initial infection, persistence, and negativisation of cervical infection and cytological diagnosis, in the cohort of 55 patients studied, Mérida- Venezuela, 2008–2012.

Cytological diagnosis		Status of HPV initial infection											Total
		Negative HPV		Positive HPV		Total	HPV individuals infections		HPV multiples infections		HPV infections by genotypes not identified		
		*n*	%	*n*	%	*n*	*n*	%	*n*	%	*n*	%	*n*
**Cytological changes suggesting of HPV infection**	**ASCUS**	1	3.45	–	–	1	–	–	–	–	–	–	–
	**LSIL**	1	3.45	6	21.43	7	4	26.67	1	14.29	1	16.67	6
	**HSIL**	–	–	2	7.14	2	1	6.67	–	–	1	16.67	2
**Cytological changes not suggesting of HPV infection^£^**		27	93.10	20	71.43	47	10	66.67	6	85.71	4	66.67	20
**Total**		29	100.00	28	100.00	57	15	100.0	7	100.00	6	100.00	28
**Cytological diagnosis**		**Status of HPV infection after follow-up for 15 +/- 5 months**											**Total**
		**Negative HPV**		**Positive HPV**		**Total**	**HPV individuals infections**		**HPV multiples infections**		**HPV infections by genotypes not identified**		
		***n***	**%**	***n***	**%**	***n***	***n***	**%**	***n***	**%**	***n***	**%**	***n***
**Cytological changes suggesting of HPV infection**	**ASCUS**	–	–	–	–	–	–	–	–	–	–	–	–
	**LSIL**	–	–	2^*^	33.33	2	–	–	–	–	2	33.33	2
	**HSIL**	–	–	–	–	–	–	–	–	–	–	–	–
**Cytological changes not suggesting of HPV infection^£^**		51	100.00	4	66.67	55	–	–	–	–	4^**^	66.67	4
**Total**		51	100.00	6	100.00	57	–	–	–	–	6	100.00	6

£: Negative for intraepithelial lesion or malignancy; ASCUS: Atypical squamous cells of undetermined significance; LSIL: Low-grade intraepithelial lesion; HSIL: High-grade intraepithelial lesion;

* new lesions reported after follow up;

** 3 HPV persistent infections.

Source: Microbiology and Public Health Laboratory, Faculty of Medicine, ULA, MPPS, Mérida.

**Table 6.** Correlation between HPV initial infection, persistence, and negativisation of cervical infection and colposcopic diagnosis, in the cohort of 55 patients studied. Mérida- Venezuela, 2008–2012.

Colposcopic diagnosis		Status of HPV initial infection											Total
		Negative HPV		Positive HPV		Total	HPV individuals infections		HPV multiples infections		HPV infections by genotypes not identified		
		*n*	%	*n*	%	*n*	*n*	%	*n*	%	*n*	%	*n*
**Colposcopic changes suggesting of HPV infection**	**Minor abnormalities**	8	27.57	8	28.57	16	4	26.67	2	28.57	2	33.33	8
	**Major abnormalities**	2	6.70	6	21.43	8	3	20.00	1	14.29	2	33.33	6
**Colposcopic changes not suggesting of HPV infection**		19	65.52	14	50.00	33	8	53.33	4	57.14	2	33.33	14
**Total**		29	100.00	28	100.00	57	15	100.00	7	100.00	6	100.00	28
**Colposcopic diagnosis**		**Status of HPV infection after follow-up for 15 +/- 5 months**											**Total**
		**Negative HPV**		**Positive HPV**		**Total**	**HPV individuals infections**		**HPV multiples infections**		**HPV infections by genotypes not identified**		
		***n***	**%**	***n***	**%**	***n***	***n***	**%**	***n***	**%**	***n***	**%**	***n***
**Colposcopic changes suggesting of HPV infection**	**Minor abnormalities**	15^♦^	29.41	3^♦♦^	50.00	18	–	–	–	–	3	60.00	3
	**Major abnormalities**	1^♦♦^	1.96	–	–	1	–	–	–	–	–	–	–
**Colposcopic changes not suggesting of HPV infection**		35	68.63	3^*^	50.00	38	–	–	1	100.00	2	40.00	3
**Total**		51	100.00	6	100.00	57	–	–	1	–	5	100.00	6

♦ Five new cases of atypical colposcopic reported after follow-up;

♦ one new case of atypical colposcopic reported after follow-up;

** three HPV persistent infections.

Source: Microbiology and Public Health Laboratory, Faculty of Medicine, ULA, MPPS, Mérida.
